# Overexpression of *StCDPK13* in Potato Enhances Tolerance to Drought Stress

**DOI:** 10.3390/ijms252312620

**Published:** 2024-11-24

**Authors:** Zhenzhen Bi, Simon Dontoro Dekomah, Yihao Wang, Zhuanfang Pu, Xiangdong Wang, Richard Dormatey, Chao Sun, Yuhui Liu, Zhen Liu, Jiangping Bai, Panfeng Yao

**Affiliations:** 1 State Key Laboratory of Aridland Crop Science, College of Agronomy, Gansu Agricultural University, Lanzhou 730070, China; bizz@gsau.edu.cn (Z.B.); dekomahsimon42@gmail.com (S.D.D.); wangyh@st.gsau.edu.cn (Y.W.); puzf@st.gsau.edu.cn (Z.P.); wangxiangd@st.gsau.edu.cn (X.W.); sunc@gsau.edu.cn (C.S.); lyhui@gsau.edu.cn (Y.L.); liuzhen@gsau.edu.cn (Z.L.); baijp@gsau.edu.cn (J.B.); 2 CSIR-Crop Research Institute, P.O. Box 3785, Kumasi 00233, Ghana; rmddormatey@gmail.com

**Keywords:** potato, *StCDPK13*, drought tolerance, antioxidant, stomatal aperture, expression analysis

## Abstract

Calcium-dependent protein kinases (CDPKs), which are activated by transient changes in the Ca^2+^ concentration in plants, are important for various biological processes, such as growth, development, defense against biotic and abiotic stresses, and others. Mannitol is commonly used as an osmotic regulatory substance in culture medium or nutrient solutions to create water-deficit conditions. Here, we cloned the potato (*Solanum tuberosum* L.) *StCDPK13* gene and generated stable transgenic *StCDPK13*-overexpression potato plants. To investigate the potential functions of *StCDPK13* in response to drought stress, overexpression-transgenic (OE1, OE2, and OE7) and wild-type (WT) potato seedlings were cultured on MS solid media without or with mannitol, representing the control or drought stress, for 20 days; the elevated mannitol concentrations (150 and 200 mM) were the drought stress conditions. The *StCDPK13* gene was consistently expressed in different tissues and was induced by drought stress in both OE and WT plants. The phenotypic traits and an analysis of physiological indicators revealed that the transgenic plants exhibited more tolerance to drought stress than the WT plants. The overexpression lines showed an increased plant height, number of leaves, dry shoot weight, root length, root number, root volume, number of root tips, fresh root weight, and dry root weight under drought stress. In addition, the activities of antioxidant enzymes (CAT, SOD, and POD) and the accumulation of proline and neutral sugars were significantly increased, whereas the levels of malondialdehyde (MDA) and reactive oxygen species (ROS), including hydrogen peroxide (H_2_O_2_) and O_2_^•−^, were significantly reduced in the OE lines compared to WT plants under drought stress. Moreover, the stomatal aperture of the leaves and the water loss rate in the leaves of the OE lines were significantly reduced under drought stress compared to the WT plants. In addition, the overexpression of *StCDPK13* upregulated the expression levels of stress-related genes under drought stress. Collectively, these results indicate that the *StCDPK13* gene plays a positive role in drought tolerance by reducing the stomatal aperture, promoting ROS scavenging, and alleviating oxidative damage under drought stress in potatoes.

## 1. Introduction

Drought stress affects many morphological and physiological processes, including the growth and yield of plants under both field and in vitro conditions [1]. This is because cell expansion and growth are suppressed by the loss of turgor pressure or an osmotic imbalance, which in turn reduces plant growth and activity of all metabolic processes [2]. Mannitol is a six-carbon sugar alcohol [C_6_H_8_(OH)_6_] that is commonly used in laboratories as an osmotic regulatory substance to control the osmotic potential in culture medium or nutrient solutions to create water-deficit conditions [3]. It is used in plants to induce osmotic stress. The high accumulation of mannitol impairs plant growth and works as a stressing agent. This stress produces drought-like condition similar to those prevailing under field conditions [4,5]. The plants which were cultured on MS medium were supplemented with the elevated mannitol (100, 200, and 300 mM) for drought stress; this can influence the gene expression, the morphological and physiological characteristics, and the biochemical content of the plants [6].

The second messenger, Ca^2+^, is a ubiquitous intracellular signaling molecule that regulates many growth and development processes [7]. When plants are exposed to various stress situations, they rapidly release calcium ions (Ca^2+^) from storage compartments (such as the vacuole and endoplasmic reticulum) into the cytosol. A transient increase in free Ca^2+^ in the cytosol is sensed and decoded by various Ca^2+^ sensors and Ca^2+^ binding proteins, such as calcium-dependent protein kinases (CDPKs), calmodulin-like proteins, calmodulins, and calcineurin B-like proteins. CDPKs differ from other calcium-sensing proteins in that they can not only decode the increase in Ca^2+^ concentration and translate it into an increase in protein kinase activity, but can also activate downstream effectors [8]. CDPKs have a conserved modular structure with a variable N-terminal domain, a kinase domain, an auto-inhibitory domain or junction domain, and a regulatory domain or CaM-like domain that canonically contains four EF-hands [9]. In the absence or low concentration of cytoplasmic Ca^2+^, the auto-inhibitory domain blocks the kinase domain and inhibits its activity [10].

CDPKs are present in protists, oomycetes, green algae, and plants, but not in animals or true fungal genomes [11]. A genome-wide study of various plant species revealed that they are encoded by a large multigene family. For example, 25 CDPKs were found in potato (*Solanum tuberosum*) [12,13], whereas there were 34 identified in *Arabidopsis thaliana* [7]; 84 and 41 were identified in cotton (*Gossypium barbadense* L. and *Gossypium raimondii*) [14,15] and 31 in rice (*Oryza sativa*) [16]. Further, a total of 20 members were confirmed in the wheat (*Triticum aestivum*) genome [17], as well as 40 in maize (*Zea mays*) [18], 29 in tomato (*Solanum lycopersicum*) [19], and 30 CDPK genes in poplar (*Populus trichocarpa*) [20]. In cucumber (*Cucumis sativus*) and grape (*Vitis vinifera*), very few CDPK members were identified, with both plants containing 19 members [21,22].

Some individual CDPKs in different plant species have been studied and assigned specific roles. For example, the overexpression of *StCDPK28* in potato enhances photosynthetic activities and drought tolerance [23], while *StCDPK2* is involved in salt stress response [24]. Likewise, *StCDPK5* and *StCDPK7* transgenic potato plants showed a high resistance capacity to the pathogen *P. infestans* [25]. The tomato gene *LeCDPK2* regulates ethylene biosynthesis in response to wound signaling [26]. The grapevine gene *VaCPK29* confers tolerance to heat and osmotic stresses [27], whereas *ZmCPK11* is involved in touch- and wound-induced pathways in maize [28]. Moreover, *OsCPK9* in rice plays a positive role in the drought, osmotic, and dehydration stress responses [29], while *OsCPK4* on the other hand, is involved in rice tolerance to drought and salt stress by protecting cell membranes from lipid peroxidation [30]. In *Arabidopsis*, *AtCPK1/2/4/5/11* phosphorylate and activate NADPH oxidase to repress the production of reactive oxygen species (ROS) in response to abiotic and biotic stimuli [31]. Similarly, CPK28 in *Arabidopsis* acts as a positive regulator in response to osmotic stress [32]. The overexpression of *SiCDPK24* in *Arabidopsis* increases drought tolerance in the transformed lines [33]. These studies indicate the important regulatory roles that CDPKs play in plant growth, development, and abiotic stress tolerance. In this study, we cloned *StCDPK13* and generated overexpression-transgenic potato plants. We analyzed the overexpression lines and wild-type control plants in terms of their gross phenotypes, physiological indicators, stomatal aperture, and stress-related gene expression under drought stress. The results support the idea that the overexpression of *StCDPK13* enhances drought tolerance in transgenic potatoes.

## 2. Results

### 2.1. Cloning and Sequence Analysis of StCDPK13

**Figure 1:**
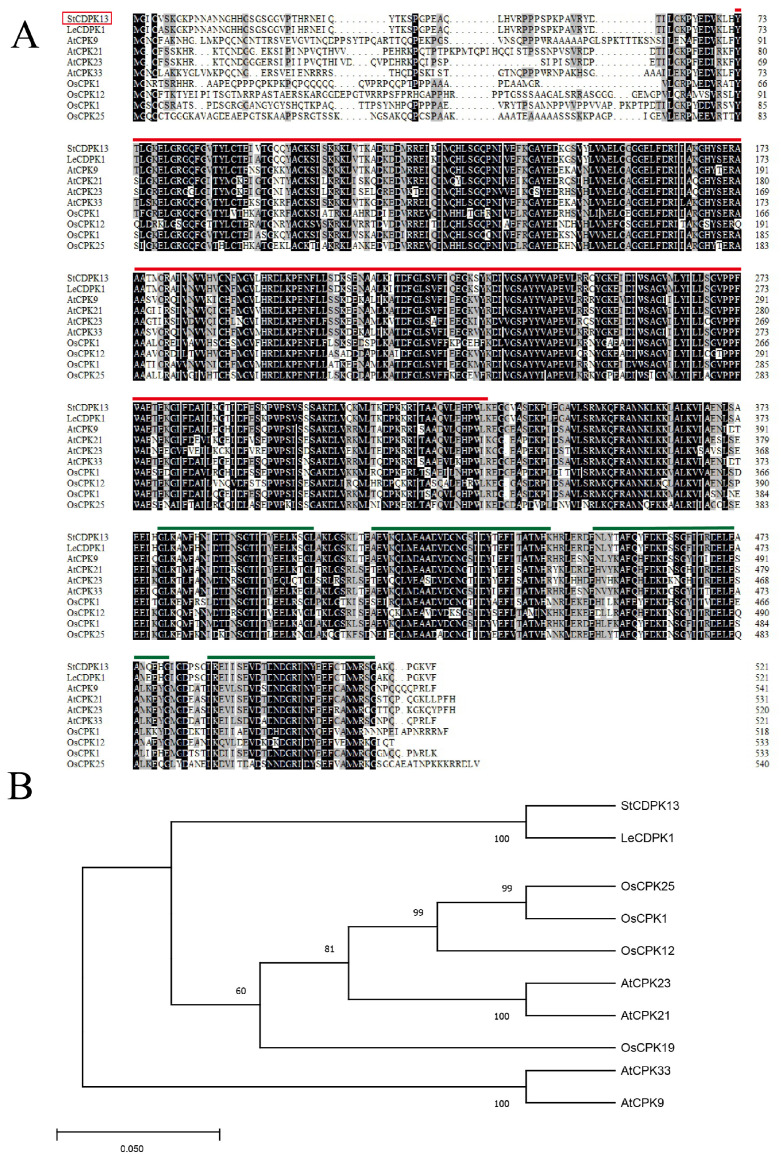
Sequence analysis of StCDPK13. (**A**) Amino acid sequence alignment of StCDPK13 with the indicated homologous CDPKs from the indicated plant species (Red line: a variable domain that harbors the ATP binding site, preceding a Ser/Thr protein kinase catalytic domain. Green lines: A CaM-like domain containing four EF hand Ca^2+^-binding motifs). (**B**) Phylogenetic tree of StCDPK13 and its homologs from different plants species (St: *Solanum tuberosum*; Le: *Lycopersicon esculentum*; Os: *Oryza sativa*; At: *Arabidopsis thaliana*). The full-length amino acid sequences of CDPKs from four plant species were aligned by DNAMAN and a phylogenetic tree was constructed from the file produced using the Maximum Likelihood method with 1000 bootstrap replicates using MEGA 5.0.

In this study, the *StCDPK13* gene (PGSC0003DMG400022318) was cloned in the AC142 potato genotype (diploid, wild-type (WT)). StCDPK13 is located on chromosome seven as the 13th gene of CDPK family, which consisted of 1566 nucleotides encoding 521 amino acid (aa) polypeptides with a predicted molecular weight of 57.86 kDa and an isoelectric point of 6.75 (Table S1). The StCDPK13 protein presents the core characteristics of a CDPK: a low complexity region (14–24 aa) in N-terminal, a variable domain which harbors the ATP binding site, preceding a Ser/Thr protein kinase catalytic domain (73–331 aa), and a CaM-like domain containing four EF hand Ca^2+^ -binding motifs (378–513 aa) (Figure 1A, Figure S1).

A phylogenetic analysis showed that StCDPK13 has a close relationship with LeCDPK1 from *Solanum lycopersicum* (*Lycopersicon esculentum*) (Figure 1B). Sequence alignment analysis of amino acid revealed that StCDPK13 shared sequence identities with LeCPKD1 of 99% (Figure 1B). Additionally, StCDPK13 was 78%, 70%, 68%, and 65% identical to OsCPK19, OsCPK12, OsCPK1, and OsCPK25 in *Oryza sativa*. It also demonstrated identical percentages with AtCPK9, AtCPK33, AtCPK21, and AtCPK23 (*Arabidopsis thaliana*) of 81%, 79%, 74%, and 66%, respectively (Figure 1A). 

### 2.2. Generation of Stable Transgenic Potato Plants

**Figure 2:**
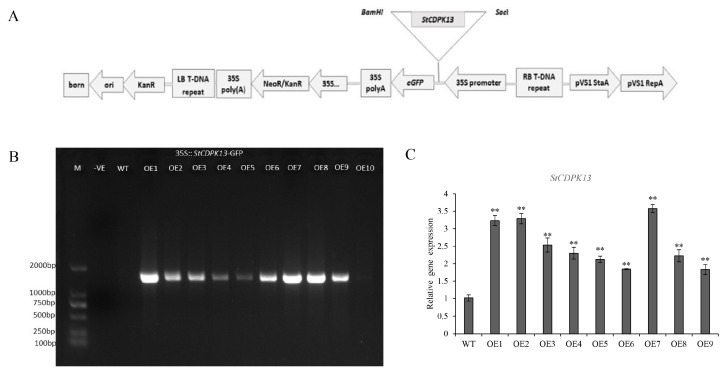
Plasmid map and identification of the positive transgenic lines. (**A**) Schematic representation of the construction of the *StCDPK13* overexpression vector. The triangular shape indicates the region of the *StCDPK13* insertion. (**B**) PCR molecular identification of positive *StCDPK13* transgenic lines. (**C**) Expression level of *StCDPK13* in positive transgenic lines under control condition. All the transgenic lines and WT plants were grown in MS media without mannitol for 20 days, and then the DNA and total RNA were extracted for PCR and qRT-PCR. ** indicate significant differences in the OE line compared with WT, based on Duncan’s Multiple Range Tests (*p* < 0.01).

To investigate the potential functions of *StCDPK13* in potato in response to drought stress, the overexpression vector of StCDPK13 (pCAMBIA2300S-StCDPK13-GFP) was constructed and transgenic potatoes were obtained via an Agrobacterium-mediated transformation (Figure 2A). Nine positive transgenic lines were confirmed by PCR, and a qRT-PCR analysis showed that the expression level of *StCDPK13* increased significantly in these transgenic potatoes (Figure 2B,C). Therefore, three independent overexpression transgenic lines (OE1, OE2, and OE7) characterized by high expression levels, were selected for further investigation.

### 2.3. The Expression of StCDPK3 in Tissues and Response to Drought Stress in Potato

**Figure 3:**
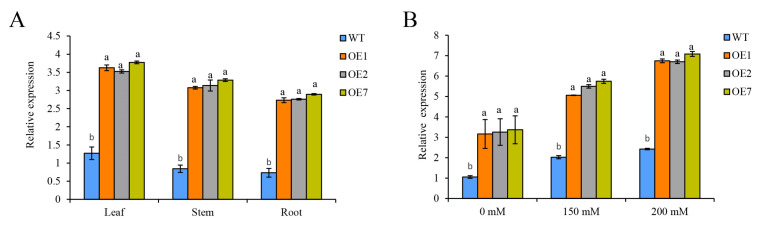
Expression profile of StCDPK13 in tissues and drought stress in OE lines and WT plants. (**A**) Tissue-specific expression of StCDPK13 in OE lines and WT plants under control conditions. (**B**) Relative expression of StCDPK13 in OE lines and WT plants under drought stress (the elevated mannitol concentrations (150 and 200 mM) are the drought stress conditions). All the values represent the means of three independent biological and technical replicates ± SE (standard error). Means with different letters indicate significant differences in OE line compared with WT, based on Duncan’s Multiple Range Tests (*p* < 0.05).

To investigate the response of StCDPK13 to drought stress in potatoes, overexpression-transgenic (OE1, OE2, and OE7) and wild-type (WT) potato seedlings were cultured on MS solid media without or with mannitol, representing the control or drought stress, for 20 days; the elevated mannitol concentrations (150 and 200 mM) were the drought stress conditions. Then, the expression levels were detected. The results showed that the StCDPK13 gene was consistently expressed in different tissues (Figure 3A). Meanwhile, the StCDPK13 gene was induced by drought stress in both the OE and WT plants, and the expression level in the OE lines was significantly higher than in the WT plants (Figure 3B).

### 2.4. Overexpression of StCDPK3 Enhances Drought Resistance in Potato

**Figure 4:**
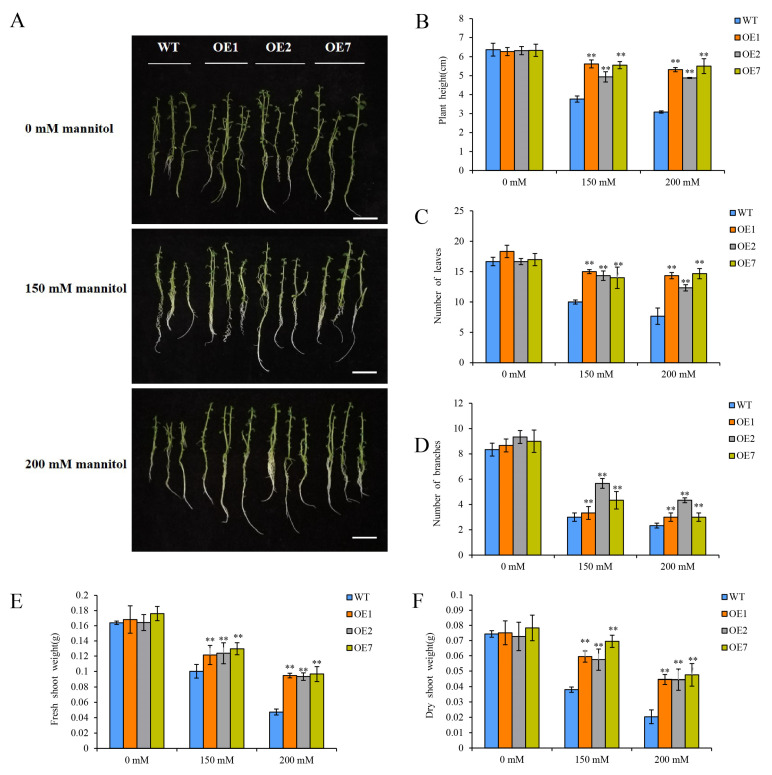
Identification of shoot phenotypic traits in OE lines and WT plants under control and drought conditions (the elevated mannitol concentrations (150 and 200 mM) are the drought stress conditions). (**A**) Phenotypes of the plants, (**B**) plant height, (**C**) number of leaves, (**D**) number of branches, (**E**) fresh shoot weight, and (**F**) dry shoot weight. ** indicate significant differences in the OE line compared with WT, based on Duncan’s Multiple Range Tests (*p* < 0.01). Bars = 2 cm.

To investigate the drought-resistance function of StCDPK13, overexpression-transgenic (OE1, OE2, and OE7) and wild-type (WT) potato plants were cultured on MS solid media without or with mannitol, representing the control or drought stress, for 20 days; the elevated mannitol concentrations (150 and 200 mM) were the drought stress conditions. Under normal conditions, there were no differences between the OE lines and the WT plants in growth and rooting. Upon exposure to drought stress, the transgenic plants showed better growth than the WT plants (Figure 4A). Further measurements of the phenotypic traits were conducted, including the plant height, the number of branches, the number of leaves, the fresh shoot weight, and the dry shoot weight (Figure 4B–F). Our findings showed that the plant height was significantly higher in the three OE lines compared to the WT plants under drought conditions. This was especially true under 200 mM mannitol, as the height of the three transgenic lines nearly reached 1.5–1.7 times that of the WT plants. Similarly, the number of leaves and the dry shoot weight displayed a comparable pattern.

**Figure 5:**
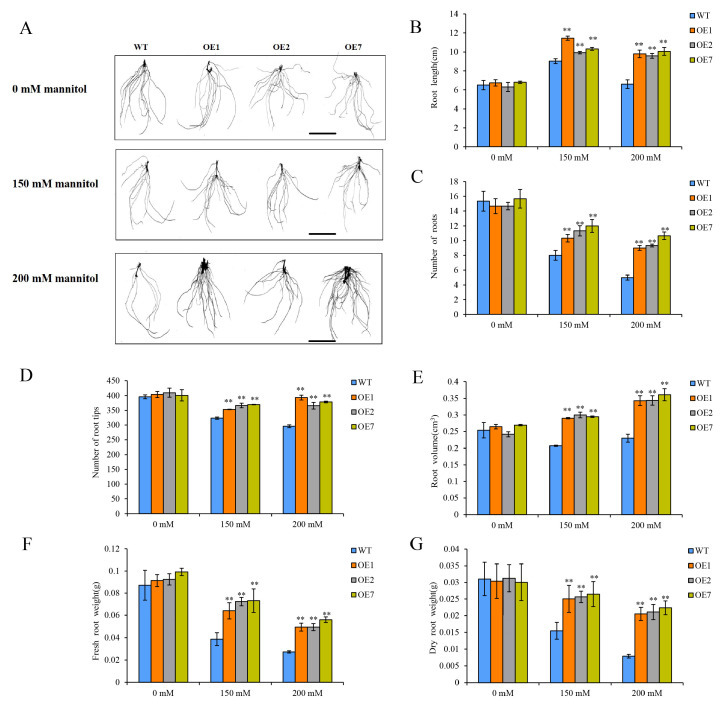
Analysis of root phenotypic indicators in OE lines and WT plants under control and drought conditions (the elevated mannitol concentrations (150 and 200 mM) are the drought stress conditions). (**A**) Morphology of root, (**B**) root length (cm), (**C**) number of roots, (**D**) number of root tips, (**E**) root volume (cm^3^), (**F**) fresh root weight, (**G**) dry root weight. ** indicate significant differences in OE line compared with WT, based on Duncan’s Multiple Range Tests (*p* < 0.01). Bars = 2.5 cm.

Meanwhile, a comprehensive assessment of the root phenotype and indicators was conducted. The root morphology of the three OE lines exhibited a superior development and tolerance compared to the WT plants under drought stress (Figure 5A). These findings indicate that the root length, root number, root volume, number of root tips, fresh root weight, and dry root weight were all significantly increased in the three OE lines compared to the WT plants under drought stress conditions (Figure 5B–G). The fresh root weight and dry root weight in the three OE lines were especially increased (by 2.1 and 2.8 times) compared to those of the WT plants under 200 mM mannitol. All these results indicate that the overexpression of StCDPK13 enhances drought resistance in potatoes.

### 2.5. Overexpression of StCDPK13 Promoted ROS Scavenging and Alleviated Oxidative Damage

**Figure 6:**
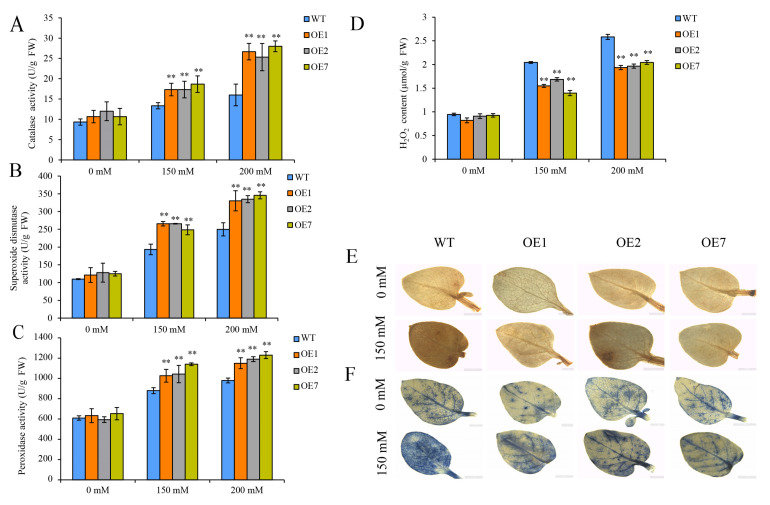
Measurement of antioxidant enzyme (CAT, SOD, and POD) activities and the levels of reactive oxygen species (ROS) in the OE lines and WT plants under control and drought conditions (the elevated mannitol concentrations (150 and 200 mM) were the drought stress conditions). (**A**) CAT activity, (**B**) SOD activity, (**C**) POD activity, (**D**) H_2_O_2_ content, (**E**) DAB staining, and (**F**) NBT staining. ** indicate significant differences in OE line compared with WT, based on Duncan’s Multiple Range Tests (*p* < 0.01). Bars = 1 mm.

Reactive oxygen species (ROS) accumulate when plants are exposed to various environmental stressors, resulting in oxidative damage and cell death [34]. Therefore, the activities of the antioxidant enzymes CAT, POD and SOD, which scavenge excess reactive oxygen species, and the content of H_2_O_2_ were measured in the three OE lines and WT plants. The results showed that the activities of CAT, POD and SOD were all increased significantly, while the content of H_2_O_2_ was significantly decreased in the three OE lines compared to WT plants under drought stress (Figure 6A–D). At the same time, we investigated the accumulation of H_2_O_2_ and O_2_^•–^ in the OE lines and WT plants under drought stress (150 mM mannitol) using DAB and NBT staining, respectively. Under the normal condition (0 mM), there were no differences between the OE lines and the WT plants for DAB and NBT staining in leaves. However, the leaves of the WT plants were more extensively stained by DAB and NBT than those of the OE lines under drought stress (150 mM) (Figure 6E,F), indicating that H_2_O_2_ and O_2_^•–^ were highly accumulated in the WT plants under drought stress.

**Figure 7:**
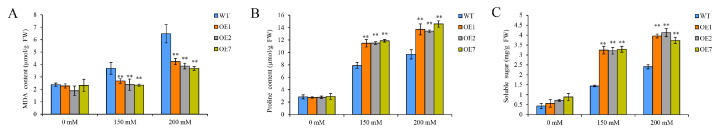
Measurement of malondialdehyde (MDA), proline, and neutral sugars content in the OE lines and WT plants under control and drought conditions (the elevated mannitol concentrations (150 and 200 mM) were the drought stress conditions). (**A**) MDA content, (**B**) proline content, and (**C**) neutral sugars contents. ** indicate significant differences in OE line compared with WT, based on Duncan’s Multiple Range Tests (*p* < 0.01).

Drought stress can cause the disarrangement of plant cell membranes. The degree of damage to plants can be reflected by the content of malondialdehyde (MDA), a final decomposition product of membrane lipid peroxidation [35]. The content of MDA was not significantly different between any of the lines under the control conditions, whereas it was significantly lower in the three OE lines than in the WT plants (Figure 7A). Proline and neutral sugars are pivotal osmolytes that can alleviate the osmotic damage caused by drought stress. We found that the proline and soluble neutral sugars levels were significantly higher in the three OE lines than in the WT plants (Figure 7B,C). These results suggest that the overexpression of *StCDPK13* can promote ROS scavenging and alleviate oxidative damage, membrane damage, and osmotic damage under drought stress in potatoes.

### 2.6. Analysis of Stomatal Aperture and Water Loss Rate of Leaves

**Figure 8:**
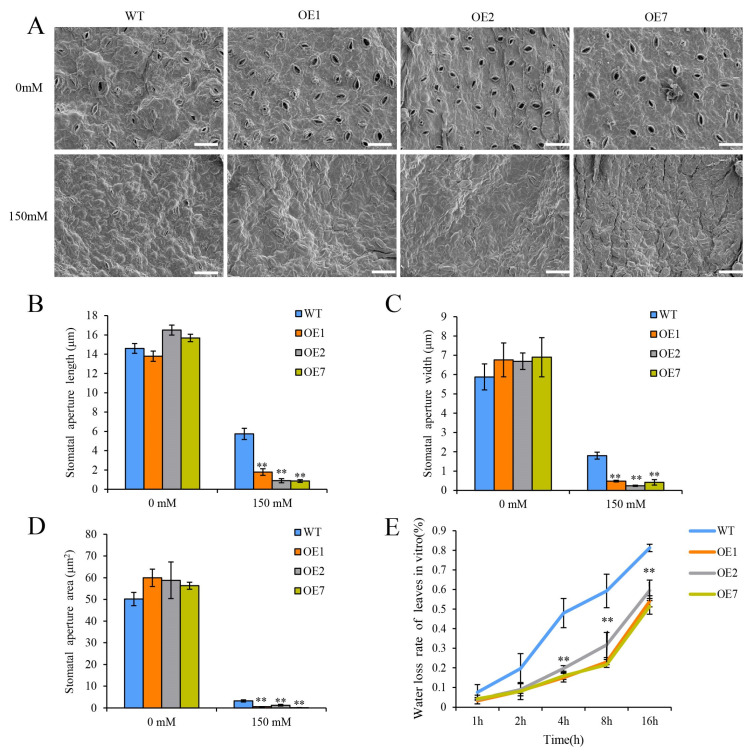
Stomatal aperture and water loss in the OE lines and WT plants under control and drought conditions (the elevated mannitol concentrations (150 mM) was the drought stress condition). (**A**) Observation of stomata in leaves under normal and drought stress conditions, (**B**) stomatal aperture length, (**C**) stomatal aperture width, (**D**) stomatal aperture area, (**E**) water loss rate of leaves in vitro. ** indicate significant differences in OE line compared with WT, based on Duncan’s Multiple Range Tests (*p* < 0.01). Bars = 20 μm.

The three OE lines and WT potato plants were cultured on MS media with 0 mM (normal) or 150 mM (drought stress) of mannitol. Then, the leaves of these plants were collected for stomatal observation. Under the normal condition, there were no differences between the OE lines and the WT plants for the stomatal aperture length, width, or area (Figure 8A). Under drought stress, there were significant reductions in all three examined stomatal aperture values in the OE lines compared to the WT plants; the stomatal aperture area of the OE1, OE2, and OE7 plants was reduced to 60%, 70%, and 69%, respectively (Figure 8B–D). Meanwhile, the water loss rate of the leaves in vitro was also determined to evaluate the amount of water evaporation. The leaves from the OE lines and WT plants were weighed at 1 h, 2 h, 4 h, 8 h, and 16 h at room temperature. We found that the leaves of the three OE lines had a significantly lower water loss rate than those of the WT plants. In particular, the water loss rate of the three OE lines was significantly lower after 2h than the WT plants (Figure 8E). These findings suggest that the overexpression of *StCDPK13* may enhance drought tolerance by decreasing the stomatal aperture and reducing water loss.

### 2.7. Expression of Drought-Rtelated Genes in StCDPK13 Transgenic Potato

**Figure 9:**
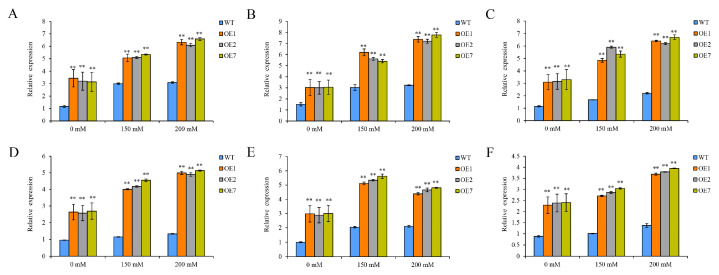
Expression levels of stress-related genes in the OE lines and WT plants under control and drought conditions (the elevated mannitol concentrations (150 and 200 mM) were the drought stress conditions). (**A**) Catalase (PGSC0003DMT400002101), (**B**) superoxide dismutase (PGSC0003DMT400042937), (**C**) low molecular weight heat-shock protein 20 (PGSC0003DMT400089913), (**D**) CAP160 protein (PGSC0003DMT400037083), (**E**) alcohol dehydrogenase (PGSC0003DMT400 063937), and (**F**) embryogenesis-abundant protein (PGSC0003DMT400021902). ** indicate significant differences in OE line compared with WT, based on Duncan’s Multiple Range Tests (*p* < 0.01).

The activation of several drought-inducible genes is an essential component of stress adaptation in plants. To investigate the potential reason that *StCDPK13* improves the drought resistance in transgenic potato plants, the expression levels of several drought-related genes were analyzed. These genes were involved in different pathways and included four drought-inducible genes (low molecular weight heat-shock protein 20, alcohol dehydrogenase, embryogenesis-abundant protein, and CAP160 protein), and two antioxidant genes (catalase and superoxide dismutase). The results revealed that these drought-related genes were all induced by drought stress, and the expression levels of all the examined genes in both the OE and WT plants showed a marked increase under drought stress compared to their levels under normal conditions (Figure 9). On the other hand, the expression levels were significantly higher in the OE plants than in the WT plants, which was expected for the embryogenesis-abundant protein. These results suggest that the overexpression of *StCDPK13* may improve drought tolerance by increasing the expression of these drought stress-related genes.

## 3. Discussion

Calcium is a second messenger in the signal transduction pathway involved in the regulation of various environmental stimuli in plants. CDPKs, which are activated by transient changes in the Ca^2+^ concentration in plants, are important for various biological processes, including growth, development, defense against biotic and abiotic stresses, and others [36]. In this study, we cloned the potato gene *StCDPK13*. Phylogenetic and alignment analyses revealed a high degree of identity between the protein sequence of *StCDPK13* and other homologous CDPKs from three different plant species (Figure 1). *StCDPK13* homologs from *Oryza sativa* (*OsCPK12*, *OsCPK9*, *OsCPK19*, *OsCPK1*) have been associated with abiotic stress responses (drought/salt) in rice [29,37,38,39]. *LeCDPK1*, on the other hand, takes part in plant defense by developing a protective mechanism against pathogens and mechanical wounding [40]. *AtCPK23*, as the homolog found in *Arabidopsis*, has been reported to play a positive role in salt stress response by controlling K^+^ channels [41], while *AtCPK9/33* negatively regulate ABA-induced stomatal closures and S-type currents [42,43]. In addition, a study by Franz indicated that *AtCPK21* is biochemically activated in response to an increased osmotic stress in vivo [44]. Meanwhile, the expression levels of *StCDPK13* were determined for different tissues and were found to be the highest in the leaves, followed by the stems; the root had the lowest transcript levels. These results suggest that *StCDPK13* may be involved in many biological functions, including growth and development (Figure 3A). In addition, the expression of *StCDPK13* was upregulated under drought conditions, in both the OE lines and WT plants. The expression levels in the OE lines were significantly higher than those in the WT plants, and significant increases were observed particularly under severe drought (200 mM mannitol) (Figure 3B). This work is similar to previous work, which found that potatoes and *Poncirus* CDPKs (*StCDPK23* and *PtrCDPK10*) were upregulated in response to drought stress [12,45], as well as studies showing the upregulation of *VaCPK29* in grapevines under heat/osmotic stress [27] and *AtCPK6* in *Arabidopsis* under drought/salt stress [46]. These results suggest the potential significance of *StCDPK13* in response to drought stress in potatoes.

Extensive studies have validated the function of CDPKs in enhancing plant drought resistance. The overexpression of *OsCPK9* [29], *OsCPK10* [47], *OsCPK7*, and *OsCDPK14* [48] significantly improved the drought tolerance of transgenic rice. Similar results have been reported in other plant species, such as *Arabidopsis* [46], maize [49], tomatoes [19], *Populus* [50], and ginger [51]. In this study, a phenotypic trait analysis revealed that transgenic plants overexpressing *StCDPK13* exhibited a higher tolerance to drought stress than WT plants. The overexpression lines showed a significantly increased plant height, number of leaves, and dry shoot weight (Figure 4). The root phenotypic indicators were also analyzed. Compared to WT plants, the root phenotype of the OE lines exhibited a superior development and tolerance under drought stress. For instance, the root length, root number, root volume, number of root tips, fresh root weight, and dry root weight were all significantly increased in the OE lines (Figure 5). These results are similar to those of a previous study showing that plants with specific root lengths are better adapted to dry conditions and have a greater capacity for water uptake [52]. Root characteristics influence a plant’s water and nutrient absorption and are important for maintaining crop yield under water stress conditions [53]. A plant’s root system is not only the site of water uptake and nutrient uptake, but also plays a significant role in the development of abiotic stress responses [54]. All these findings indicate that the overexpression of *StCDPK13* enhances drought tolerance.

Drought stress leads to water deficiency, the inhibition of plants growth, and the accumulation of reactive oxygen species (ROS) in plants, such as H_2_O_2_ and O_2_^•−^. The excessive production of ROS results in oxidative damage and eventual cell death [34]. Under drought stress, the expression levels of genes (such as *CAT*, *SOD* and *POD*) that encode antioxidant enzyme were upregulated, resulting in the increases of antioxidant enzyme activity (CAT, SOD and POD) [34]. An increase in antioxidant enzyme activity promotes the scavenging of ROS and the establishment of a protective mechanism to diminish the exposure to oxidative damage in plant cells [55]. There are several indications that overexpression of stress-inducible CDPK genes may improve drought tolerance in plants by scavenging ROS or upregulating osmolyte accumulation [56]. This study found that under drought stress, the activity of CAT, SOD, and POD was significantly increased, and the content of H_2_O_2_ was significantly decreased in the OE lines compared to WT plants. The 3,3-diaminobenzidine (DAB) and nitroblue tetrazolium (NBT) staining also showed an extensive accumulation of H_2_O_2_ and O_2_^•−^ in WT plants under drought stress (Figure 6). These results are similar to reports stating that the overexpression of *OsCDPK4* in rice improved the drought/salt stress tolerance by protecting cell membranes from stress-induced oxidative damage [36], while *OsCPK12* reduced ROS accumulation by increasing the expression of *OsAPx2*, *OsAPx8*, and *OsrbohI*, conferring salt tolerance in rice [50]. Drought stress can also cause the disarrangement of plant cell membranes. The degree of damage to plants can be reflected by the content of MDA, a final decomposition product of membrane lipid peroxidation [35]. Tobacco plants carrying apple *MdCPK1a* are more resistant to salt and cold due to the fact that they produce lower levels of MDA [57]. Moreover, proline and neutral sugars are pivotal osmolytes that can alleviate the osmotic damage caused by drought stress. Transgenic *Arabidopsis* lines with *AtCPK6* showed reduced MDA accumulation and an increased proline content under drought stress conditions [46]. Similarly, our studies found that the overexpression of *StCDPK13* exhibited a reduced MDA content and increased the accumulation of proline and neutral sugars under drought stress (Figure 7). These results suggest that the overexpression of the *StCDPK13* gene promotes ROS scavenging and alleviates oxidative damage, membrane damage, and osmotic damage under drought stress in potatoes.

The ability to store water is important for plants to survive drought stress. The stomata of leaves play an important role in the water loss of plants, and water vapor loss in mature leaves depends on the forced evolution of plant gas exchange capacity and stomatal size and density [58]. Plants with less water loss are more tolerant under drought stress. Previous studies have reported that water loss in plants is explicitly linked to the stomatal aperture. The overexpression of *ItfWRKY70* enhances drought tolerance by promoting stomatal closure, leading to a higher content of water [59]. A knockdown of *AtCPK3* and *AtCPK6* lead to a more open of stomatal aperture and increase drought sensitivity [60] whereas the overexpression of *AtCDP4* and *AtCPK11* enhances drought tolerance through ABA induction of stomatal closure under drought stress. In this study, we found that the leaves of the OE lines exhibited smaller stomatal apertures and a significant decrease in the water loss rate of leaves compared to WT plants under drought stress (Figure 8). These results indicate that the overexpression of *StCDPK13* improves drought tolerance by regulating the stomatal movement.

Studies have reported that low molecular weight heat-shock protein 20 plays an important role in maintaining membrane stability and detoxifying ROS by regulating antioxidant enzymes in plants under stress, particularly heat or drought stress [61]. In a similar report by Zhao et al. [62], low molecular weight potato heat-shock protein 20 was upregulated under heat stress. The increased expression of this gene in the OE lines could be the reason for their increased tolerance to mannitol-induced drought stress. Meanwhile, alcohol dehydrogenase has been examined, which is simultaneously responsible for catalyzing the reversible conversion of acetaldehyde to ethanol and the oxidation of NADH to NAD+ to enable plant survival under stressed conditions [63]. Moreover, under stress conditions, embryogenesis-abundant proteins are induced to confer membrane and protein stability, osmotic regulation, and serve as a protective buffer against dehydration [64]. Moreover, the CAP160 protein, which showed strong upregulation in the OE lines, was identified as drought-sensitive in potatoes such as Longshu3 and Tajfun cultivars [65,66]. In other plants, such as pepper and spinach, the CAP160 gene expression was upregulated in response to water deficit [67], which is consistent with our study data. This study found that the expression of the drought-responsive genes low molecular weight heat-shock protein 20, alcohol dehydrogenase, embryogenesis-abundant protein, and CAP160, were all significantly increased in OE lines compared to WT plants under drought stress (Figure 9C–F). In addition, the expression of catalase, and superoxide dismutase genes was upregulated in the OE lines under drought stress as opposed to control plants (Figure 9A,B). These findings are consistent with those of Dong et al. [57], who found that transgenic *MdCPK1a* tobacco lines exhibited the upregulation of antioxidant-related genes under stress, as well as the high expression level of antioxidant-related genes in a drought-tolerant potato genotype (Qingshu9) reported by Wang et al. [68]. The upregulation of drought-related genes in the overexpression lines of *StCDPK13* could potentially explain their protective roles in potatoes under drought stress.

## 4. Materials and Methods

### 4.1. Plant Materials and Growth Conditions

Potato seedlings of the AC142 genotype (diploid, wild type, WT) and transgenic potatoes overexpressing *StCDPK13* were used in this study. The potato seedlings were micropropagated in culture bottles on a modified MS medium without or with mannitol, representing the control or drought stress. The elevated mannitol concentrations (150 and 200 mM) were the drought stress conditions. Five seedlings were cultivated in each culture bottle and fifteen replicate bottles were examined for each line and each treatment. The culture bottles were cultivated in an artificial growth chamber under a long day photoperiod (16/8 h light/dark) at 23 °C for 20 days. Phenotypic traits, including the plant height, the number of leaves, the number of branches, the fresh shoot weight, the dry shoot weight, the root length, the root number, the root volume, the number of root tips, the fresh root weight, and the dry root weight were determined. Shoots were collected and measured for physiological indicators and gene expression. These physiological indicators included the activity of CAT, SOD, and POD, and the content of H_2_O_2_, MDA, proline and neutral sugars. The genes included *StCDPK13* and drought-related genes. All the assays were performed using three independent biological replicates (seedlings from three bottles), each with three technical repeats.

### 4.2. Cloning and Sequence Analysis of StCDPK13

To analyze *StCDPK13*, multiple amino acid sequences of StCDPK13 were aligned with the DNAMAN 9.0 software (LynnonBiosoft, San Ramon, CA, USA). A phylogenic tree was constructed using the MEGA 5.0 software (https://www.megasoftware.net/, accessed on 15 March 2021) with the neighbor-joining method. The total RNA of the potato plants was extracted using an RNAprep Pure Plant Kit (Tiangen Biotech, Beijing, China), and first-strand cDNA synthesis was performed using a ReverTra Ace^®^ qPCR RT Master Mix with gDNA Remover (TOYOBO, Osaka, Japan), following the protocol of the kit. The full length CDS (1566 bp) of *StCDPK13* was amplified by RT-PCR with the following specific PCR primers: forward 5′-ATGGGTATTTGTGTTAGTAAAGGT-3′ and reverse 5′-GAAGACCTTGCCTGGTTATTTGGC-3′, using the high-purity enzyme KOD FX.

### 4.3. Obtaining Transgenic Potato Plants

The synthesized cDNA was used as a template for StCDPK13 amplification, with the specific PCR primers forward 5′-(*SacI*) ATCTTGCTGAATACCCATCTGTGT-3′ and reverse 5′-(*BamHI*) AATGTGGTGAAATCTTCGACGA-3′. The PCR product was SacI and BamHI restriction enzymes and was ligated into the pCAMBIA2300-GFP vector to generate the recombinant plasmid pCAMBIA2300-StCDPK13-GFP. The construct was then transformed into Agrobacterium tumefaciens GV3101 cells using the heat-shock method. The recombinant plasmid was then transformed into a diploid potato control plant (AC142) using the A. tumefacien-mediated method according to the procedure of Grandellis et al. [38]. The regenerated transgenic seedlings were cultivated on an MS medium supplemented with hygromycin (50 mg L^−1^, *w*/*v*). Positive lines were then confirmed using PCR and the expression level of StCDPK13 was analyzed using qRT-PCR.

### 4.4. Phenotype Analysis of WT and Transgenic Plants Under Drought Stress

StCDPK13-overexpression and WT potato plants were cultured on MS medium with 0, 150, or 200 mM mannitol for 20 days. Then, the phenotypic and physiological indicators were determined. The number of leaves and branches were recorded from each plant in all the treatments. The plant height was measured with a meter rule at the time of sampling. Several root indices, including the root length, the root number, the root volume, number of root tips, the fresh root weight and the dry root weight were measured on both stressed and unstressed plants using a Root Scanner (STD) 4800 (EPSON, Quebec City, QC, Canada) and the root image analysis software Win RHIZO version 5.0 (Regent Instruments, Inc., Quebec City, QC, Canada).

### 4.5. Determination of Physiological Indicators Under Drought Stress

The catalase (CAT) activity was assessed according to Hamurcu et al. [69], where one unit of CAT activity was considered the amount of enzyme required to decompose 1 µmol of H_2_O_2_ at 240 nms^−1^ in 1 mg of fresh tissue protein. The peroxidase activity (POD) was determined according to Huang et al. [70]. The superoxide dismutase (SOD) activity was determined by measuring its ability to inhibit the photochemical reduction of nitroblue tetrazolium (NBT), and the change in absorbance was measured at 560 nm [71].

The total hydrogen peroxide content (H_2_O_2_) of the leaves was assessed according to the method of Junglee et al. [72]. H_2_O_2_ and O_2_^•−^ were identified by staining the leaves of drought-stressed plants with 3,3-diaminobenzidine (DAB) and nitroblue tetrazolium (NBT), as previously described by Kong et al. [73]. The contents of proline and malondialdehyde (MDA) in fresh shoots were measured according to the method of Bates et al. [49]. The contents of neutral sugars were determined using the anthrone colorimetric method [68].

### 4.6. Analysis of Stomatal Aperture and Water Loss Rate in Leaves

Leaves detached from WT and transgenic plants were fixed in 2.5% glutaraldehyde at room temperature for 2 h and at 4 °C for 24 h, and dehydrated with 30%, 50%, 70%, 90%, and 100% ethanol and tert-butanol. They were then vacuum dried, pasted, and sprayed gold, and the stomata were imaged using a tungsten-filament scanning electron microscope (SEM, Hitachi-S 3400N, Tokyo, Japan). The images were used to estimate the stomatal apertures with the help of the ImageJ software (downloaded from https://imagej.nih.gov/ij/download.html, accessed on 1 February 2022) according to the method of Sun et al. [59]. About 40 stomatal pores from the same region of each leaf were examined for each measurement assay.

The water loss rate of the leaves was also measured. Leaves were detached from the OE-line plants and WT plants grown on an MS medium without mannitol for 20 days and placed on filter paper. The leaves were weighed at 1 h, 2 h, 4 h, 8 h, and 16 h. The water loss rate was calculated as [(FWo − FWi)/FWo × 100%], where FWo is the original fresh weight and FWi is the fresh weight at a specific time interval [6]. These tests were conducted in a controlled environment at 22 °C.

### 4.7. Expression Analysis of the StCDPK13 Gene and Stress-Related Genes

The total RNA of the OE lines and WT plants which were cultured on MS media without or with mannitol for 20 days, was extracted and reverse-transcribed to synthesize cDNA. The transcript levels of *StCDPK13* in the leaves, stems, and roots were measured for the OE lines and WT plants under normal conditions. The expression of the *StCDPK13* gene and stress-related genes in the leaves of the OE lines and WT plants under drought stress were assessed with qRT-PCR using specific primers (Table S2). All the qRT-PCR assays were performed using three independent biological replicates, each with three technical replicates.

### 4.8. Data Analysis

All the experiments were performed for three independent biological replicates with three technical repeats. The data were analyzed using the SPSS software, version 16.0 software (SPSS Inc., Chicago, IL, USA), for statistical evaluations with the general linear model (GLM). All the data are presented as the mean ± S.E. (standard errors). Post hoc comparisons of the means were performed using Duncan’s Multiple Range Test (DMRT), to assess the variability among the three overexpression lines (OE) and wild-type (WT) plants with the statistical significance defined as *p* < 0.05 (*) or *p* < 0.01 (**).

## 5. Conclusions

In this study, we cloned a member of the CDPK gene family in potato, *StCDPK13*, and found that its overexpression in potato enhanced drought tolerance by reducing the stomatal aperture, promoting ROS scavenging, and alleviating oxidative damage. *StCDPK13* plays a positive role in drought tolerance and may offer a valuable approach for improving the drought tolerance of potato.

## Data Availability

Data are contained within the article and Supplementary Materials.

## Supplementary Materials

The following supporting information can be downloaded at: https://www.mdpi.com/article/10.3390/ijms252312620/s1.
