# Supplementary material for: Overexpression of StCDPK13 in Potato Enhances Tolerance to Drought Stress
Source: Int J Mol Sci. 2024 Nov 24;25(23):12620. doi: 10.3390/ijms252312620 (PMC11640904; doi:10.3390/ijms252312620)
Supplement: Supplementary file 1 [file ijms-25-12620-s001.zip › Figure S1. Amino acid sequence analysis of StCDPK13.pdf]

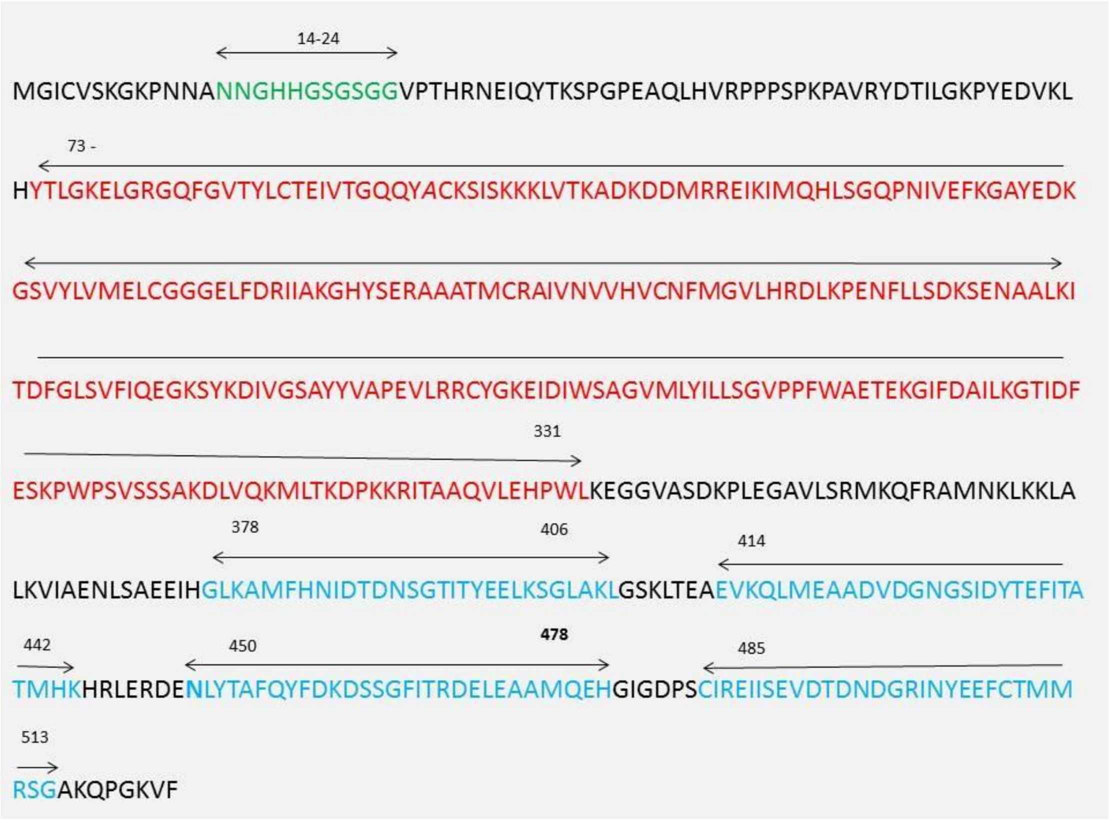

Figure S1. Amino acid sequence analysis of StCDPK13. Low complexity region is highlighted in green from 14 – 24 amino acids (aa). The Ser/Thr protein kinase catalytic domain is shown in red from 73 – 331 aa, containing the active site, ATP binding site, polypeptide substrate binding site, and the activation loop (A - loop). The EF hands location is colored in blue: EF hand I (378 -406 aa), EF hand II (414 – 442 aa), EF hand III (450 – 478 aa), and EF hand IV (485 – 513 aa).
