# Supplementary material for: Overexpression of StCDPK13 in Potato Enhances Tolerance to Drought Stress
Source: Int J Mol Sci. 2024 Nov 24;25(23):12620. doi: 10.3390/ijms252312620 (PMC11640904; doi:10.3390/ijms252312620)
Supplement: Supplementary file 1 [file ijms-25-12620-s001.zip › Table S2. The sequences of primers were used for qRT-PCR.pdf]

Table S2. The sequences of primers were used for qRT-PCR.

Genes	Sequences
StCDPK13	F: 5'-TTGGGAAGGAGTTAGGA AGAGG-3'<br>R: 5'-CATCTTCCACATCTATCTCTGTTCTC-3'
Catalase	F: 5'-AGTGCCTGCTCATGAAGTGG-3'<br>R: 5'-TGGAGAGAAATCATGGCCCAG-3'
Superoxide dismutase	F: 5'-TTCACTCCCC GATCTCCCAT-3'<br>R: 5'-GCTGACAGGG GCAAGGTTAT-3'
Low molecular weight heat-shock protein 20	F: 5'-AGAAACCCCGGAAGCTCATG-3'<br>R: 5'-CATTCTCCATCGCC GCCTTA-3'
CAP160 protein	F: 5'-CACCAA CAGGCGTAGTGGAT-3'<br>R: 5'-CGG GCTCCTGTATCGGTAAC-3'
Alcohol dehydrogenase	F: 5'-CCAGCAATGCCGTCACTGTA-3'<br>R: 5'-TGC CTGTGGATCGATCTTGG-3'
Embryogenesis abundant protein	F: 5'-CTGGTGGCACTG GTGGAAAA-3'<br>R: 5'-CCGCCTTTT CGACCCATTTC-3'
Actin	F: 5'-AGGAGCATCCTGTCCTCCTAA-3'<br>R: 5'-CACCATCACCAGAGTCCAACA-3'
